# Some Account of the Febrifuge Bark of a Tree Called El Malambo, the Produce of South America

**Published:** 1833-06

**Authors:** Wm. Hamilton


### TRANSACTIONS OF THE MEDICO-BOTANICAL SOCIETY OF LONDON.

*Published under the Direction of the President and Council.*

### FEBRIFUGE BARK OF EL MALAMBO.

*Some Account of the Febrifuge Bark of a Tree called El Malambo, the Produce of South America.* By Wm. Hamilton, M.D. (Communicated in a Letter to Earl Stanhope, President of the Med.-Botanical Society.)

In the latter part of the year 1825, I received from my correspondent, Mr. Watts, his Britannic Majesty's consul at Carthagena, in Columbia, some samples of a febrifuge bark, called el Malambo, the produce of a tree with which I am wholly unacquainted. The samples consisted of pieces of the bark in substance, and an ounce of it in powder. As it reached me by way of Liverpool, and the carriage of the bark in substance would have been attended with considerable difficulty and expense, I requested a friend there to place this last at the disposal of some medical gentleman likely to do justice to its investigation, while the powder was transmitted to me by post. The bark in substance was accordingly given to Dr. TRAIL; of the result of whose trials I have never since been favoured with any report; and the powder I transmitted to Dr. BOSTOCK, whose clinical researches pointed him out as a person likely to promote the objects I had in view. After some delay, I was favoured by him with the following letter, dated London, 11th December, 1825.

"Sir: I regret that so long a time should have elapsed since the receipt of your letter. When it arrived I was far from home, and have since that time been very particularly engaged. The account which is given of the bark is certainly very much in its favour, and its sensible properties are such as to render it probable that it may be a useful article of the materia medica. The only plan, however, to establish a point of this nature would be to send a quantity of the substance to some hospital, or dispensary, where a number of trials might be made of it by any person in that kind of extensive practice. Its taste and odour would make me suppose that it may be found useful in affections of the stomach and bowels, as is the case with colombo or angustura; and, even if its properties were found to be very similar, still it might be important to have a variety of articles. As I have no connexion with any medical charity, it will not be in my power to give you any assistance, and I think it will be best for you to apply to some practitioner in your neighbourhood.

"I am, sir, your most obedient,

"J. BOSTOCK."

Mr. Watts, in his letter, dated Carthagena, 19th July, 1825, says,

"I wish particularly to draw your attention to a valuable production of this province: it is the bark of the Malambo tree. I enclose you a little of the powdered bark; and Mr. Byrne has charge of a parcel containing some pieces of the bark. You have also enclosed a description of this bark, and its qualities, given me by Don Jorge Lopez, the apothecary, my neighbour, with a translation. I shall be glad to hear what opinion may be formed in England of this bark, and whether it is likely to become an article of exportation from this port; for it can be procured in any quantities. I enclose also another description of the Malambo tree, extracted from another rare and valuable Spanish work in my possession, with a translation.

The following are the accounts referred to:

" *Descripcion del Malambo, por Don Jorge Lopez.*

" Sedia, en la provincia de Cartagena, un arbol de la misma altura de los Quinos, nombrado Malambo, cuja corteza en mí concepto tiene qualidades mas recomandables que la Quina: ella abunda de un aromatico tan constante y penetrante que jamas lo pierde, y por supuesto abunda tambier de partes gomosas muy utiles, à mi ver, à la naturaleza; es indigena de esta provincia, y se encuentra con abundancia. Su corteza as quehabla, y crea que si se dedicase los facultativos à hacer usa de ella, la Quina perderia su concepto. En este pais la aplican à diversos males con ventaja conocida, en particular para los pasmos, heridas, inflamaciones, males del estomago; a mi ver producivia un efecto admirable en la hidropesia, y fiebres malignas; por varias observaciones que se ban hecho es effeax para las asmas, reumatisma, disenteria, supresiones de menstruas; en fin, en el pais, y en particular en los campas, es un remedia general aplicado con felix suceso en todas las enfermedadas referidas."*

" *Descripcion del Malambo, (extracted from a rare Spanish work.)*

" El tercero es el Malambo, cuja corteza aromatico es un ànti-spasmodico eminente, de un amargo muy activo, febrifugo, y astringente. Lo hay con abundancia en la provincia de Santa Marta, donde lo aplican para curar pasmo, calenturas, intermittentes, y la dysenteria; y tambien se encuentra en varios territorios de esta provincia. Se hacen algunas extracciones de su corteza par la Habana, y alli lo usan con mucha suceso contra el pasmo, à que son muy propensas los negros; y *desde que tien este specifico casi no muera ninguno deel.* Aqui no se aplica para nada, y hace pocos dias hemos visto morir un joven apreciable, par no haberle dado en tiempo esto remedio. Es un vegetal importantissimo, que merece la atencion del gobierno por las virtudes que dexamos expuestas, y aplicaciones que se hacen de el en Santa Marta; como

* *Translation of the Description of the Malambo, given by Don George Lopes.*

There is found in the province of Carthagena a tree of the same height as the Quinas, (bark-trees,) named the Malambo, the bark of which, in my opinion, possesses more valuable qualities than even the Quina. It abounds with an aromatic so strong and so penetrating, that it never loses it under any circumstances; and, of course, it likewise abounds in gums highly useful, in my idea, to humanity. It is indigenous to this province, and is found in great abundance. It is to the bark of the tree which I allude, and I really believe that, were the faculty to make use of it, the Quina (Peruvian bark) would fall in their estimation. In this country it is applied to several diseases with well-known success; in particular, in cases of spasms, wounds, inflammations, diseases of the stomach. In my opinion, it would produce the happiest results in cases of dropsy and malignant fever. From several other observations, it is also found highly efficacious in asthmas, rheumatisms, dysentery, and suppression of the menses. In short, in the province, and particularly in the country parts, it is a remedy applied with the happiest success in all the cases enumerated above.

y por color y olor de su corteza, tenemos razones para creer sea del genero Cusparia, ò Quina de la Angostura, de que se compane parte del receta del difunta Dr. Mutis, para curacion de la dysenteria, y na duda mos aseguar que, a falta de esta, puede stiplir la corteza del Malambo."*

Such is the account given by the late canon Don Jose Ignacio del Pombo, the friend and pupil of the lamented and learned Mutis, in his rare and almost-unknown work on the Natural Productions of the Province of Carthagena.

Having requested a further supply of Malambo bark for experiment, I at length received a second supply of some pounds, in the summer of 1827, at a time when indisposition had confined me for some months to the house. I in consequence applied to my friend Dr. Cookworthy, whose practice at the dispensary pointed him out as a person likely to promote my views, especially as he had facilities for getting it reduced to powder, which were not within my reach; and I placed the whole quantity, with the exception of one piece, (fragments of which accompany this paper,) at his disposal. The bark was in pieces of from a foot to eighteen inches in length, about two inches in breadth, and three eighths of an inch in thickness; the outer and thinner coat was externally of an ash-colour, marked with irregular longitudinal furrows, and separating from the inner or thicker coat, which had more of a ligneous texture, or any attempt at breaking the bark, by bending the fragment marked *a*, exhibits both the inner and outer coats of the bark united; on fracture, the smell was agreeably aromatic, and the taste not unpleasantly bitter. I failed in my expectations of obtaining a satisfactory report of its effects from Dr. Cookworthy, and could only learn from him, in conversation, that it was as effectual in arresting the progress of intermittents as the cinchona; but

* *Description of the Malambo, (extracted from a rare Spanish Work.)*

The third is the Malambo, the aromatic bark of which is a powerful antispasmodic, a very active bitter, febrifuge, and astringent. It abounds in the province of Santa Martha, where it is applied to the cure of spasm, intermittent fever, and dysentery; it is likewise found in several districts of this province. It is sent (the bark) in some quantity to the Havana, and there it is used with much success in cases of spasms, to which the negroes are very subject; and since they are in possession of this specific scarcely any die of it. Here no use whatever is made of it, and not many days ago we have seen a very amiable young man carried off, from not having taken this remedy in time. It is a most important vegetable, and deserving the attention of government, for the virtues we have enumerated, and the use made of it in Santa Martha. From the colour and odour of its bark, we have reason to believe it is of the genus Cusparia, or Quina of Angustura, of which is in part composed Dr. Mutis' receipt for the cure of dysentery; and we have no doubt that the want of this might be supplied by the Malambo.

the bulk of the dose rendered its exhibition objectionable, especially since the introduction of quinine, a few grains of which produced all the effects of as many ounces of either the Peruvian or Malambo bark. I urged him in vain to get his brother to analyse a portion, and determine the nature and quantity of its active constituents; and I found that, by trusting to others, I had again lost the opportunity which this second supply afforded me of investigating its useful qualities. I had, however, retained one piece of the bark, which I treasured up, in the hope of some future opportunity offering for getting it analysed; and this piece I shall gladly place at the disposal of the Society.

I was equally unsuccessful in my applications to Mr. Watts for specimens of the plant which produced it; and hence I am unable to throw the smallest light upon the genus to which it belongs, or advance anything beyond the merest conjecture. That it does not belong to any of the true cinchonas, which differ from the Exostemas in having their stamina included, seems probable from HUMBOLDT's observation, (*Pers. Narr.* vol. iii. p. 29, *note*,) that almost all the cinchonas of the inferior regions are Exostemas, not true cinchonas, the properties of which differ considerably from those of the genuine cinchonas of the Andes; while from his observation of the fact of his not having found a single specimen of Exostema during his long abode on the coast of Cumana and Caraccas, the banks of the Apuri, the Oronoko, and Rio Negro, in an extent of country of four thousand square leagues, it seems equally improbable that it belongs to the genus Exostema. It is probable therefore that it belongs either to the febrifuge bark-tree described by Dr. HANCOCK, the Cusparia of the banks of the Oronoko, the Machaonia, the Cuspa of Humboldt, a nondescript plant, the inflorescence of which Humboldt has never been able to procure, notwithstanding its blossoming at the end of November, and being abundant in the province of Cumana; or perhaps a new and distinct genus.

Humboldt describes his Cuspa, which, he says, must by no means be confounded with the Cuspare (Cusparia febrifuga) of Angostura, as a tree of not more than fifteen or twenty feet in height, with alternate, oval, entire, leaves, destitute of stipules, and differing in this respect from all the trees of the family of Rubiaceæ, to which the Cinchonas and Exostemas belong, and yielding a very thin pale-yellow bark, possessing more bitterness, but at the same time less disagreeable, and agreeing better with the stomach than that of the true cinchona, and possessing great febrifuge powers. This description by no means corresponds with the Malambo bark, which is rather thick, of a cinereous colour, agreeable to the smell, and of a bitterness not only less, but totally different in taste, than the bark of any of the cinchonas. It also appears, from the account of Don Ignacio de Pombo, to be a powerful and valuable antispasmodic; and the fact of its having, from the first period of its introduction into Cuba, completely checked the mortality previously so frequent among the blacks, from the attacks of a spasmodic disorder to which they were subject, "*desde que tien este specifico casi na muere ninguna de el,*" is an important recommendation, which merits further investigation.

Possibly it might even be found of service in arresting the progress of hydrophobia, some of whose most distressing symptoms are evidently of a spasmodic character. Spasm, the term applied by the prior to the complaint so prevalent, and formerly so fatal, among the negroes of Cuba, is so vague a denomination as to leave us in a state of uncertainty as to the true character of the complaint; but tetanus, to which negroes are often subject, is probably the disorder to which he refers, and is one which bears considerable analogy to hydrophobia; and it is this circumstance which suggested to me the possibility of this bark being useful, if only as a palliative, in this latter and still more melancholy affliction.

I cannot sufficiently regret that my information does not enable me to furnish anything in the shape of positive information, beyond the statements furnished by the prior and the Carthagena apothecary, whose exaggerated description is only calculated to provoke a smile. Should circumstances ever permit of my visiting the regions which produce it, I hope to be able to lay before the Society more precise intelligence respecting it, accompanied by a botanical description and specimens.

Postscript. I have no distinct recollection of having ever communicated to your lordship a striking instance of the powerful effects of the seeds of the Hibiscus Abelmoschus, or Musk Ockra, in counteracting the fatal influence of the bite of venomous reptiles, related to me in one of his letters by Mr. Watts, our public-spirited consul at Carthagena, as having fallen within the immediate limits of his own observation; I shall therefore repeat the facts of Mr. Watts' communication here.

A peasant, resident on the heights of La Papa, near

Carthagena, being engaged with his dogs in pursuit of a hare, a dog, the best of his number, had the misfortune to be wounded by one of the most venomous snakes of that district. The dog instantly dropped, foamed at the mouth, and the wounded part began to swell. The peasant, regarding the case as desperate, at first resigned the dog to what he imagined to be his inevitable fate; but returning home, and recollecting that the animal had been a favourite, and the best of his pack, he determined to revisit the spot, and ascertain the degree of hope to be entertained. On reaching the spot, he found the animal stretched out, unable to move, and evidently in great agony: taking him up in his arms, he hurried home with him, and immediately rubbed the swoln and wounded part well with the bruised seeds of the musk plant, or Almisdenia, as the Hibiscus Abelmoschus is called there; forcing a considerable quantity at the same time down the animal's throat, with such success, that symptoms of amendment rapidly appeared, and the dog, at the date of Mr. Watts' letter, was perfectly recovered; although nothing is more certain than that his death would have been inevitable, had he been left to his fate. Yet here the application was made under circumstances by no means favourable, since much time had necessarily been lost, and the symptoms of constitutional affection from the action of the poison had actually begun to exhibit themselves.

The Musk Ockra is a common plant in most of our West-India islands, and its seed could be easily obtained from thence to almost any amount. It certainly merits further investigation, and will, I trust, attract the notice of some of the scientific members of the Society. A saturated tincture of the bruised seeds would, in all probability, be the most convenient form for internal exhibition.

